# A comparison of assessment tools for childcare centers in high vs. low resource settings

**DOI:** 10.3389/fpubh.2024.1331423

**Published:** 2024-11-13

**Authors:** Hy V. Huynh, Eve Puffer, Jan Ostermann, Andrew K. Weinhold, Meghan E. Lopez, Melissa McGovern, Kathryn Whetten

**Affiliations:** ^1^Center for Health Policy & Inequalities Research, Duke Global Health Institute, Duke University, Durham, NC, United States; ^2^Department of Psychology and Neuroscience, Duke University, Durham, NC, United States; ^3^Arnold School of Public Health, Health Services Policy and Management, University of South Carolina, Columbia, SC, United States; ^4^Johns Hopkins University School of Nursing, Baltimore, MD, United States; ^5^School of Social Work, University of North Carolina at Chapel Hill, Chapel Hill, NC, United States; ^6^Sanford School of Public Policy, Duke University, Durham, NC, United States

**Keywords:** childcare centers, low resource settings, childcare assessment tool, quality of childcare, child development

## Abstract

Studies suggest issues may arise when using childcare setting assessment tools designed for high-resource settings in low-resource settings to assess and improve the quality of care, including placing disproportionate weight on features of the childcare environment that may not be available or culturally appropriate within the low-resource context. This study compares a novel assessment tool developed in and for low-income and low-resource settings with a standardized “gold standard” tool developed for use in high-resource settings. The study included a randomized sample of 34 childcare centers in a low-resource context that provided care for approximately 918. Results suggest that the WCI-QCUALS assessment tool performed better at differentiating among childcare settings that were consolidated into the lowest rating on the ECERS-R. Further, the WCI-QCUALS was found to be a feasible, appropriate stand-alone tool for assessing the quality of childcare centers in low-resource settings. Additional refinement and validity testing in other countries and contexts will improve the understanding of how the quality of childcare across different contexts can be measured, and improved assessment of childcare quality in low-resource settings will enhance the ability to identify low-quality care that can be remedied to ensure appropriate care for vulnerable children.

## Introduction

Higher quality of childcare is recognized to be predictive of a range of positive developmental outcomes for children including cognitive functioning, emotional adjustment, language development, and social competence (1–4, 34). While there is agreement that quality of care matters for children, and there are validated tools for measuring quality of childcare in high-income and high-resource (5, 6), there are few measures available that are designed to assess differences in quality of care for childcare centers in low- and middle-income and -resource settings (7, 35). Childcare assessment tools are useful for identifying environments that support appropriate developmental growth and allow for identifying areas of competence and areas for improvement (8, 9, 31). A significant issue with utilizing an early childhood care and education assessment tool designed in a high-resource setting and applying it in a low-resource setting is the potential bias the original tool may have toward features of the childcare environment that may not be available, accessible, or culturally relevant within the low-resource context (10, 36, 37). The result of using such tools in low resource settings could be that environmentally safe childcare centers with excellent caregiver-child interactions, for example, are rated poorly because of lack of material items. An improvement plan for such a center would be costly and would not be helpful in improving the cognitive and emotional development of the children in its care. The use of such assessment tools may be the reason why little variation in childcare setting ratings in low resource settings are identified, with the majority of ratings indicating that the quality of care is poor. Multiple studies in low- and middle-income countries using environmental rating scales developed in high-resource settings, such as the HOME Inventory (e.g., 37–39) and the Infant Toddler and the Early Childhood Environmental Rating Scales [ITERS, ECERS; e.g., (8, 11)], were found to detect little variation at the low end of the scale (30). Another recent study highlighted limitations for the use of the ECERS in Colombia, stating low scores and limited variability (12). While these studies do not suggest that these measures are not valuable, they point to the need for further innovation in assessment and perhaps tailored assessment to different types of context.

Consequently, these tools are perceived by many child development workers in low-resource settings as being unhelpful and ineffective for identifying areas for meaningful intervention and improvement (30). Additionally, from an implementation perspective, such existing assessments often came with licensing and training costs in addition to standard implementation costs, rendering them generally inaccessible for many low-resource contexts. Further, results from these tools may result in allocating limited resources predominantly toward material improvements, which may not yield as significant an impact on child outcomes as investments toward improving the quality of the caregiver-child interactions. Additionally, there is a paucity of information on how to assess quality of care accurately and consistently for varying cultural contexts in ways that can meaningfully inform programs and policies.

A group (13) working in concert with the government of Nicaragua to improve the well-being of children in childcare settings confirmed what had been seen in other low- and middle-income countries. The team used the ECERS and ITERS for pre- and post-intervention assessments (11, 27, 28). On an overall scale from 1 to 5 for care setting quality, with 1 being the lowest, the average from three centers was 1.6, with little variation around the mean (11). The care setting intervention, which focused primarily on improving the caregiver-child interactions, resulted in children’s well-being improving 15.5 developmental quotients (DQs) points on the Battelle Development Inventory (32), whereas before the intervention 82% of the children had DQs <70 and only 27.8% had DQs <70 4 months and 17 months post-intervention. Yet the ITERS and ECERS rating scores rose only to a mean of 1.83 (14). The group in Nicaragua created a new assessment tool that prioritized caregiver-childcare interactions, safety, locally available and accessible toys and materials and nutrition (37).

The government of El Salvador requested assistance in assessing, improving and monitoring the governmental childcare centers across the country from the NGO Whole Child International. The government and NGO requested funding for this initiative from the Inter-American Development Bank which provided support and requested that the gold-standard ECERS assessment tool be used for the intervention. The El Salvadorian team expressed concern that the assessment tool, developed within and for high resource environments, would inadequately assist them in identifying areas of concern and overlook areas of strength. Therefore, this substudy was supported to simply compare results from the gold standard assessment tool of childcare centers used extensively in high and low-resource settings, the ECERS, with an assessment tool developed within and for a low-resource setting, called the Whole Child International Quality Childcare Universal Assessment for Limited Resource Settings (a.k.a WCI-QCUALS; available upon request from the creators) (15). Systematically examining how the quality of childcare environments is measured across contexts can help us take a step forward in providing appropriate assessment of childcare environments where fewer material resources are available. This can provide meaningful data that can be used to help guide childcare centers toward the improvement of child outcomes and to drive policy decisions related to the most vulnerable populations of children that are informed by evidence.

In this sub-study, three Salvadoran childcare environment raters from the research team with previous training and expertise in child psychology were trained and officially certified in using the ECERS-R. These raters had previous training and experience with the WCI-QCUALS. The side-by-side comparison of rating results provide early exploratory findings to generate hypotheses for future, formal validation testing.

### Study setting: El Salvador

Deprivation and poverty are common for children in El Salvador, with 41% of households with children under 18 years old being poor, compared to a rate of 24% within adult-only homes (16). In urban and rural areas, the percentages are 45 and 59.8% respectively, and El Salvador has the second highest rate of children living in precarious housing conditions in Latin America (17). Malnutrition indicators are also high, with 14% of children under five suffering from chronic malnutrition (18). Additionally, very high levels of community-level violence in El Salvador further increased the risk for children’s physical and mental health issues (15, 34). Despite unmet needs related to children’s well-being, there is a limited public contribution to early childhood development services.

The public childcare system is administered by the Salvadoran Institute for Comprehensive Child and Adolescents’ Development (ISNA) and is composed of urban Child Development Centers (CDI); rural and peri-urban Comprehensive Wellbeing Centers (CBI) which provide daily childcare; and residential Protection Centers (CDA), which provide protection to marginalized children who are not living with their families and serve as orphanages. According to the most recent estimates, there are 16 CDIs, 190 CBIs, and 10 CDAs in El Salvador, serving a total of approximately 7,000 children not including those in private centers (about 1,237 in CDIs, 4,894 in CBIs, and 350 in CDAs) (19). These numbers are inadequate for multiple reasons. First, childcare centers play a valuable role in supporting better child development outcomes, including school readiness; only 2.2% of children have access to early childhood education (20), and there is significant attrition starting at age nine (21). Increasing the volume of childcare settings in El Salvador is vital in reaching the Sustainable Development Goal of 1 year of pre-primary education (22). Further, these care settings allow women to work outside of the home, improving household income and resources for supporting children. In addition to this limited supply, the country currently does not have an appropriate assessment of the quality of the centers that serve this population or policies on quality standards for these centers (19).

### Study methods: comparison of the WCI-QCUALS with an assessment tool designed in high resource settings

At the request of the Salvadoran Government and with funding from the Inter-American Development Bank, Whole Child International (WCI) worked in collaboration with researchers from the University of El Salvador and Duke University to initiate an assessment of government-assisted residential care settings for children in El Salvador. The research team conducted an independent review of childcare and school rating instruments found to be most highly associated with positive child development in high-income countries and identified the ECERS-R (23) to be a gold standard in such environments. The Inter-American Development Bank had several projects in low and middle income countries in which the ECERS-R was used and supported the use of this instrument in El Salvador. However, child welfare government officials believed that an assessment tool that was developed by child development experts from and in low and middle income countries would likely find more helpful variation in child care settings and thereby provide a greater ability to detect areas of feasible improvement for the childcare centers. The new evaluation provided the opportunity to use and compare two assessment tools: the WCI-QCUALS and the ECERS-R. The overall aim of this sub-study was to conduct a preliminary, descriptive comparison of results from the two assessment tools in a low-income country and context. Agreement across instrument domains that intend to assess similar characteristics was examined providing the opportunity to describe unique information that may be generated by each of the tools. This information was intended to generate ideas related to the strengths and limitations of the assessment tools, especially related to their use in low-resource settings, and to guide future use and potential adaptations for new contexts.

### Study sample of care settings

This study included a randomized sample of 34 childcare centers, including 1 CDI (located in an urban area) and 33 CBIs (located in rural and peri-urban areas), all of which provide daily non-residential care for children living elsewhere in the community. The child care centers varied significantly by size and type of location, ranging from those serving 10 children with three staff in rural areas to those serving 78 children with seven staff in urban areas. In total, the sample of centers provides care for approximately 918 children with 105 staff. Centers also varied on other potentially important characteristics, including sources of funding, infrastructure, and levels of external support from the community (local government, churches, families, etc.). It is also worth noting that CDIs and CDAs are staffed by government-employed salaried caregivers, whereas CBIs are typically staffed by women from the local community who receive a government stipend for their work. Anecdotal evidence also suggests disparities in funding and material support between urban and rural centers, but this was not a focal point of the current evaluation.

### Study procedures

All procedures for the overall study were approved by the University of El Salvador Institutional Review Board and The José Simeón Cañas Central American University (a.k.a. Universidad Centroamericana, or UCA). Evaluators participated in separate trainings on the administration of both assessment tools. For the WCI-QCUALS, training of evaluators was conducted in person by WCI senior staff who were involved in the development of the tool. For the ECERS-R, the intervention funders were able to pay for three evaluators to complete the required online certificate course created by the Environmental Rating Tools Institute, which included training modules and exams resulting in the interviewers being officially certified to administer the ECERS-R. The training on both tools was supplemented with observed practice in childcare settings in El Salvador. Training is required for both assessment tools, as they are evaluator-administered tools that use some specialized language and require an understanding of the child development context in which they are administered (33). It is also important to note that both tools involve some subjectivity in assessments, so training is crucial for building a shared understanding and consistency.

Using paper workbooks and tablets with the mobile application, the ECERS-R and the WCI-QCUALS were conducted concurrently in the sample of 34 childcare centers by two different teams of interviewers. Each team of consisted of at least 2 interviewers with their assessments later evaluated for consistency and reconciled if differences were found. Following all study initiation and informed consent procedures for the overall trial, evaluators conducted assessments using the ECERS-R and WCI-QCUALS between March and May 2015 The ECERS-R was conducted on paper using the standardized workbook for the tool. The WCI-QCUALS was implemented electronically using Google Nexus tablets with a secure data transfer and storage system (iSEE) developed at Duke University. Evaluators visited a given center in groups of three to collect data over 3 days. The direct observational measures of childcare setting quality were conducted on the first day’s visit, which was unannounced, allowing for observations to be conducted under operating conditions that were as normal as possible. At each center, one rater completed the ECERS-R and at least one separate rater completed the WCI-QCUALS. At a subset of the centers, two or three raters all completed the WCI-QCUALS independently to allow for some level of assessment of inter-rater reliability on the new measure. At least one administration of the WCI-QCUALS observation focused on the same group of children in the center while the ECERS-R was collected, allowing for comparisons across instruments in the same setting.

#### Rating and scoring procedures

Items on the WCI-QCUALS assess indicators across the 10 domains identified through the initial development steps (Table 1).

**Table 1:** Ten core domains of assessment in the WCI-QCUALS.

Domain	Indicators
Administration	*Caregiver Schedules, Communication Between Staff at all Levels, Effectiveness in Implementation, Resource Management, Concept Management, Staff Management*
Environment	*Indoor/Outdoor Materials, Gross Motor, Fine Motor, Types of Spaces, Flow/Design, Accessibility*
Small Groups	*Numbers, Mixed Age, Gender, & Ability, Siblings, Temperament, Inclusion*
Continuity of Care	*Consistency of Caregiver with Child, Daily Activities, Scheduling of Caregiver, Daily Transitions, Major Transitions*
Primary Care	*Assignment, Documentation Journals, Birthday Celebrations, Appointments*
Freedom of Movement	*Flow/Center Design, Spaces Available, Activities Available, Gross Motor Options, Use of Restrictive Materials, Time Outside of Crib, Daily Schedule*
Interactions between Caregiver and Child	*Discipline, Eye Contact, Physical Approach, Dialog, Volume/Tone/Pitch, During Routines, During Free Play*
Attachment to Caregivers	*Reaction to Caregivers, Reaction to Non-Caregiver Staff, Reaction to Strangers, Child Engagement of Caregiver*
Health and Hygiene	*Water, Food Preparation, Cleanliness of Indoor/Outdoor Environment, Staff Training, Health & Hygiene Policies, Preventative Health Care, Illness and Disease Management*
Safety and Security	*Disaster Preparedness, Safety Policies, Staff Training, Toys, Environment, Confidentiality, Security*

The first four sections of the tool are designed to gather basic information via existing center records and interviews with center leaders. The following General Information section builds a basic profile of the center, including contact information, populations served, safety procedures, and access to utilities, among other things. Subsequently, the Interview section includes an in-depth structured survey conducted with at least one individual at each center: (a) director or administrator, (b) professional-level staff or supervisor, or (c) direct caregiver. These interviews focus on the quality indicators: routine center practices, procedures, and norms related to the 10 assessment domains. Child Demographics collects details on each child present to paint an aggregated picture of age, sex, time in the center, groupings, vaccination status, and educational status. Staff Demographics follows a similar structure, but instead focuses on each staff member’s role in the center, previous work experience, work/vacation hours, and current group assignment.

The fifth section includes a series of direct observations ideally conducted by two independent raters. Observations include a walk-through of the entire center and one-hour observations of childcare routines and activities. Specific observation items also map onto the 10 domains; however, given that observation items are designed to relate to more than one of the 10 domains, the observations are organized into four components: General, Environment (primarily structural and material), Relationship (primarily characteristics of direct caregiver-child interactions), and Identity (primarily related to children’s opportunities to see themselves, and for others to see them, as unique individuals). Within each of these categories, several subtopics are organized into scoring units. For each scoring unit, the rater replies to 2–8 multiple-choice questions until enough information is collected to determine a score based on what they have observed. Both the in-depth interviews and observational components are designed using skip patterns to maximize efficiency. Broad questions are presented first and based on the response, raters either continue or skip to relevant questions. In the Observation section, this typically means that questions first ask about characteristics that could trigger the lowest score (e.g., “Did any communication take place during the routine?”), then move on sequentially to more specific, higher-scoring questions (e.g., “Was the routine used as a relationship-building moment, rather than just a moment to complete a task?”). The electronic survey automatically skips to the next set of questions once a score is reached, thus avoiding the inefficiency of displaying unnecessary questions to the interviewer. Additionally, the electronic administration of the measure allows for more efficient data cleaning, and programs written by the study team eliminate the need for any manual scoring.

The information from all the assessment sections above is compiled to generate quantitative ratings across the 10 domains (Table 1). Each domain ultimately receives an overall rating between 1 (most negative) and 7 (most positive) that incorporates usable information from all five sections of the WCI-QCUALS. These ratings are then combined to generate one overall center score, also on a scale of 1 to 7. Domain weights are based on the quantity of evidence identified during the literature review that supports the importance of that practice or standard in determining overall quality. Scores are established through intermediate weights to allow for the weighting of some items or domains as more impactful than others, and for the weighting of observational versus interview and records review data. This is one of the important and unique characteristics of the WCI-QCUALS, as the weights were designed to explicitly address concerns about the limitations of scoring systems in existing tools. Different weights are also used for different types of childcare settings based on whether children reside in the setting full-time, as some practices differ in their relevance depending on whether the child spends all or only part of their time in the setting. Calculations of overall domain scores account for these weights across scoring units to arrive at the final score. In turn, the ratings on the 10 domains also are weighted before generating the overall center score. These procedures and associated calculations are described in further detail in the full WCI-QCUALS manual (24).

#### Quality improvement recommendations

After scores are generated across the 10 domains for a given center, automated recommendations that correspond to the score received for each domain are provided in a center-specific report. Like the assessment items, these are generated from the best practices identified during the literature review. Therefore, the recommendations are very similar to the definitions of the higher scores on the measure. That is, a center with a low score on a domain—for example, Freedom of Movement—would receive suggestions that provide steps to reach the criteria for a higher score in Freedom of Movement, such as creating more open play spaces and altering schedules for the youngest children to spend more time outside of cribs.

#### Mobile application: administration and scoring

For the intervention study described in the second half of this paper, a mobile application was developed for both the administration and scoring of the WCI-QCUALS. The WCI-QCUALS can be delivered and scored via paper by trained interviewers; however, the mobile application has several advantages over paper versions, including automated skip patterns, automated scoring and weighting calculations, and no need for any *post-hoc* manual data entry. The application was developed for use on Android devices.

### Data analysis

For the comparison between the WCI-QCUALS and ECERS-R results, we calculated and compared domain scores and total center scores for both assessment tools. We first examined the distributions of the total scores across the two tools to compare overall variability and trends reflecting whether one tool resulted in higher or lower scores overall. Second, we examined descriptive statistics from the two tools independently to observe the general patterns of scores for individual domains. Third, we examined the items within the domains of each tool more closely to generate lists of items and domains on which most centers received low scores and on which most received high scores. This was exploratory and aimed to gain a preliminary understanding of the types of items and constructs on which centers in this context performed better or more poorly for each tool.

From these observations, items were identified on the assessment tools that had conceptual overlap—that is, items that aimed to assess the same types of characteristics of the setting. This was an important and somewhat difficult process given that the WCI-QCUALS and ECERS-R categorize items differently and both have unique items that do not have similar counterparts on the other tool. The extent to which scores on items that did and did not overlap measured items that are contextually relevant in El Salvador and childcare centers in other LMICs were examined. Patterns emerged related to the types of items that could explain any overall differences in performance across the assessment tools, particularly those on conceptually similar constructs. The research team then discussed the results, alongside field observations from the staff and raters in El Salvador, to generate hypotheses about the reasons for differences, to make observations about the strengths and limitations of each measure, and to develop suggestions for future uses and potential adaptations of the tools; these are presented in the Discussion section.

## Results

### Comparison of total score distributions

**Figure 1:**
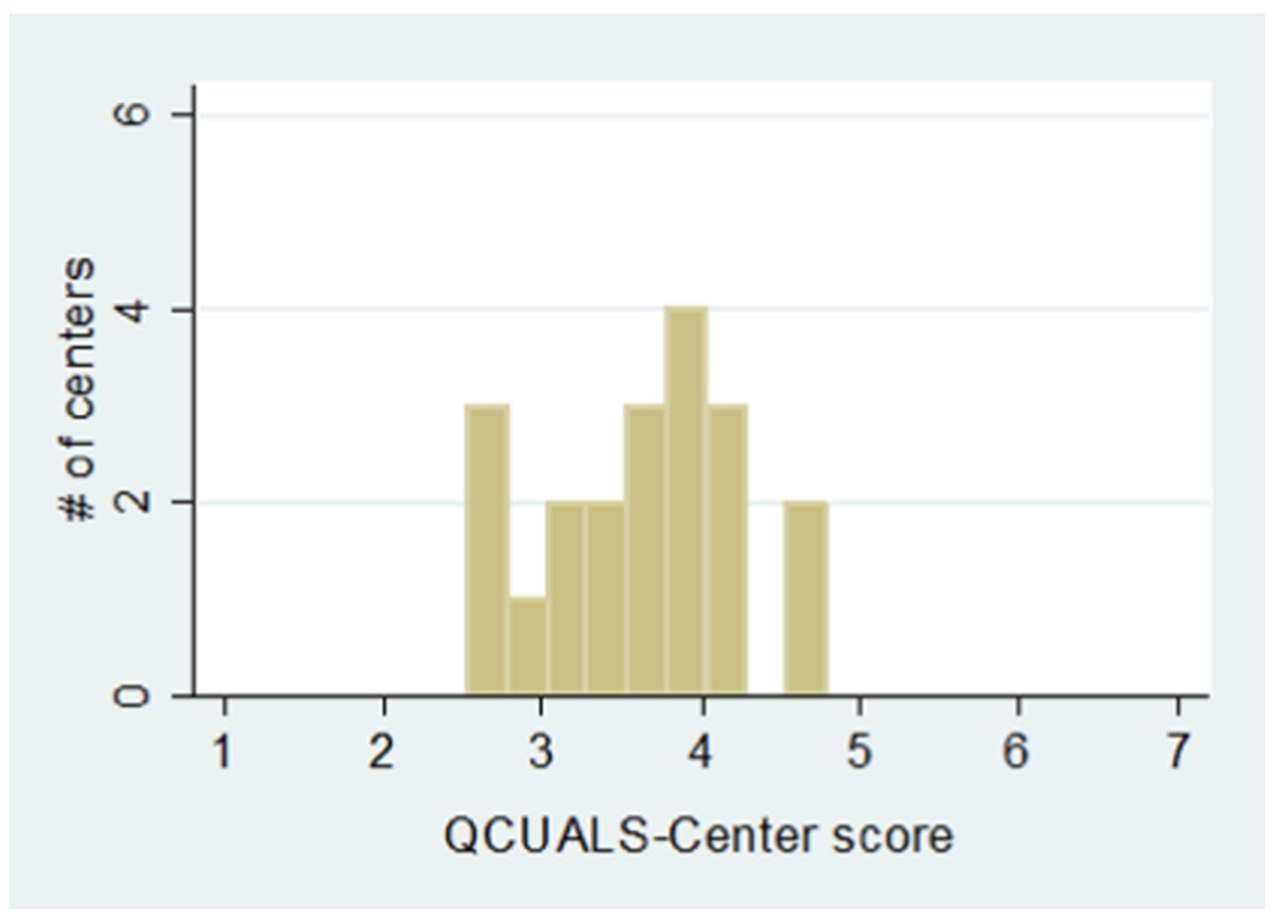
QCUALS rating score range.

**Figure 2:**
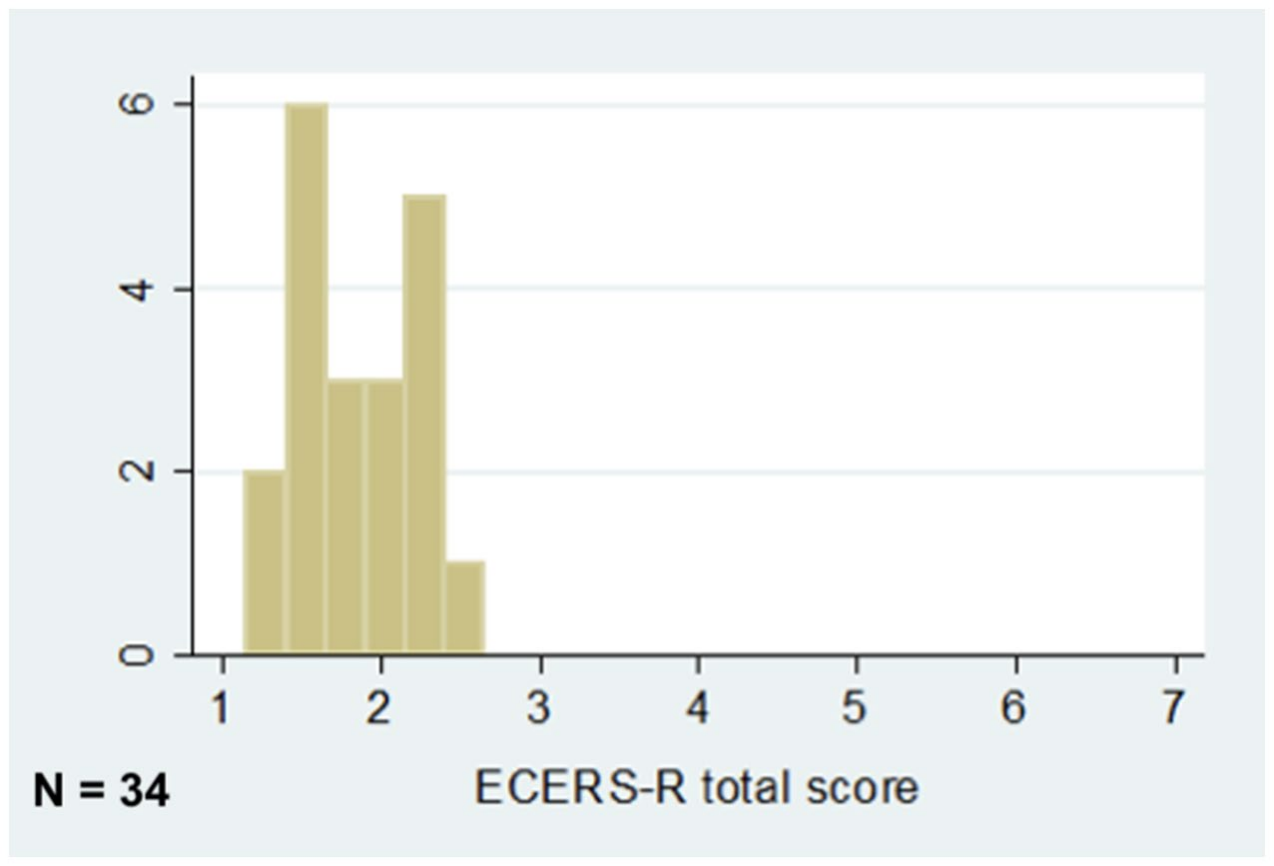
ECERS-R QCUALS rating score range.

Centers’ ratings on the WCI-QCUALS and the ECERS-R are described in Figures 1, 2. Both scales range from a minimum possible score of 1 (worst possible performance on all items) to a maximum possible score of 7 (best possible performance on all items). WCI-QCUALS scores (mean 3.6; SD 0.60; range 2.5–4.7) were centered around the midpoint of the range, whereas ECERS-R scores (mean 1.8; SD 0.39; range 1.1–2.6) were concentrated on the lower end of the range. The correlation coefficient of 0.610 (*p* = 0.004) indicates a moderate-to-strong correlation between centers’ overall ratings with the two tools without considering variation on subscales.

### Exploring domain-specific scores for each assessment tool

#### WCI-QCUALS

Across domains, ranges reflect that scores spanned the rating scale, from 1.5 to 7.0 (Table 2). Many centers had lower scores on Environment than on the other domains. This domain includes items that assess indoor and outdoor space and materials, such as the availability of soft spaces, organization of materials, ambient noise, and how conducive the overall environment is to ideal development, movement, and play. Items on which scores were particularly low, pulling down scores in this domain, including those focused on the materials present in the physical environment, such as lack of outdoor furniture and materials for play and the presence of safety hazards.

**Table 2:** Center ratings on 10 core domains of the WCI-QCUALS.

*Domain*	*Mean (sd)*		*Number of items*	*Range (1–7 possible)*
1. Administration	4.2	(0.8)	34	[2.9–6.5]
2. Environment	3.1	(0.8)	24	[1.5–4.7]
3. Small Groups	4.0	(1.2)	6	[1.5–6.6]
4. Continuity of Care	5.1	(0.8)	13	[3.3–7.0]
5. Primary Care	4.1	(1.0)	7	[2.1–6.2]
6. Freedom of Movement	4.7	(1.0)	13	[2.6–6.4]
7. Interaction Between Caregiver and Child	4.3	(1.1)	16	[2.1–7.0]
8. Attachment to Caregivers	5.4	(0.7)	7	[4.0–7.0]
9. Health and Hygiene	4.4	(0.6)	26	[2.8–5.5]
10. Safety	4.4	(0.9)	8	[2.9–7.0]
Total WCI-QCUALS Score	4.5	(0.4)	154	[3.5–5.6]

In contrast, the highest scores were on items included in the Attachment to Caregivers category, which assesses children’s reactions to caregivers, non-caregiver staff, and strangers. Scores were particularly high on items related to interactions with non-caregiver staff and previous care within the childcare system. There were also items with significant variability within this domain that seem to differentiate between centers, including whether children display indiscriminate affection to strangers. Another domain with overall high scores was Continuity of Care, which focuses on the consistency of interactions and routines (e.g., supervision, transitions between activities, and caregiver scheduling). Items with the highest scores included the number of caregivers per shift and having a consistent center schedule or routine. Like the Attachment category, there were also items with helpful variability, including the child’s movement through groups at the center, supervised free play, and caregivers performing routines in the same order. Interestingly, these two domains on which centers earned the highest scores are among the most unique to the WCI-QCUALS given the detail with which aspects of caregiver-child interactions are assessed. Therefore, it is likely that the inclusion of these domains, in addition to the inclusion of non-material items relevant in low- and middle-income countries and contexts (LMICs; Table 3), is driving the overall higher scores on the WCI-QCUALS compared with the ECERS-R.

**Table 3:** QCUALS non-material items not covered in ECERS-R.

Children do not demonstrate indiscriminate affection toward strangers
Children’s personal history not shared in group settings
Eating area cleanliness
Children are in observable good health
Information about the child is documented
Ambient noise (indoor/outdoor)
Freedom of movement
Introduction of visitors
Staff interactions

#### ECERS-R

Table 4 shows the ratings across domains for the ECERS-R. Overall, the range of item scores also was mostly restricted from 1.0 to 5.0 and showed no attainment of domain scores at the upper end of the spectrum. As shown, many centers had lower scores on Personal Care Routines and Activities than on the other domains. The Personal Care Routines domain includes items that assess the greeting and departing process, meals and snacks, nap and rest time, toileting and diapering, health practices, and safety practices. Low item scores on toileting and diapering, along with safety practices, pulled down the domain score in this instance. These items did seem to be relevant in a low-resource setting, without much concern about bias, and are therefore likely useful in identifying relative weaknesses in these settings. The Activities domain, on the other hand, received particularly low scores on items that could be considered better suited for an assessment of a classroom in a high-income country. Items such as sand and water play, dramatic play, and mathematics all frequently contributed to low scores that may unfairly count against the centers due to their minimal relevance in this particular setting. This reflects a general emphasis that the ECERS-R places on availability and control over materials and physical space, which can be resource-dependent in contexts like El Salvador and may be less relevant in LMICs (Table 5).

**Table 4:** Center ratings on ECERS-R subscales.

*Domain*	*Mean (sd)*		*Number of items*	*Range (1–7 possible)*
1. Space / Furnishings	1.8	(0.5)	8	[1.1–3.1]
2. Personal Care Routines	1.4	(0.6)	6	[1.0–3.3]
3. Language- Reasoning	1.8	(0.9)	4	[1.0–4.3]
4. Activities	1.5	(0.3)	10	[1.0–2.3]
5. Interaction	2.1	(1.2)	5	[1.0–5.0]
6. Program Structure	1.9	(0.8)	4	[1.0–3.7]
7. Parents and Staff	2.6	(0.6)	6	[1.4–3.8]
Total ECERS-R Score	1.8	(0.4)	43	[1.2–2.6]

**Table 5:** ECERS-R items less relevant to low- and middle-income/resource contexts.

Temperature control, sound-absorbing materials, natural lighting control
Woodwork bench, sand/water table, or easel
Wall-to-wall carpeting
Child-sized toilets and sinks
Rotating play materials
Sand/Water play
Dramatic play
AV/Video/Computers
Promoting acceptance of diversity
Staff lounge
Separate office space for staff

## Discussion

While there is agreement that quality of care matters for children, most assessment tools to measure the quality of childcare were developed in and designed for high-income and high-resource settings (5, 6), leaving few measures available to assess the quality of childcare settings in lower-resource settings. This study compared a gold standard assessment tool of childcare centers used extensively in high-resource settings with an assessment tool developed within and for a low-resource setting. The ECERS-R, while not intended by its creators for use in middle- and low-income countries, is among the most widely used childcare assessment tool in low and middle income countries (8). Results from this study suggest that a tool developed in low-resource area, such as the WCI-QCUALS assessment tool, a tool developed and designed in and for lower-resource childcare settings with child care experts from that country, performed better at differentiating quality of care among childcare settings that were consolidated into the lowest rating on the ECERS-R. More specifically, this is possibly due to the WCI-QCUALS inclusion of domains such as Attachment to Caregivers and Continuity of Care, and more non-material items relevant to LMICs, such as variety in toys in texture, size and color that are locally made and not as many of those items as is expected in a high resource area. At the same time, the ECERS-R places a more general emphasis on availability and control over materials and physical space, which may be more resource-dependent aspects related to quality of care. For example, providing toilets of a certain height and kind is costly and perhaps not as important as other interactive features in the environment. Further assessment tool refinement, validation, and testing are needed to improve our understanding of how to assess the quality of care more accurately and consistently for varying cultural contexts in ways that can meaningfully inform programs and policies.

Although this study was meant to generate hypotheses, it also acknowledges several limitations. Training for the ECERS-R and the administration of the tool was cost-prohibitive for the group, which only allowed three staff members to be trained in it. This limitation is important because the government was only able to pay for those three staff members who were trained by the US based rating team and developers due to the external grant that they received. The cost of training raters and then to use the tool, in itself, can be prohibitive and not necessarily a wise use of limited resources when in a low resource environment. The sample’s geographic locations were also very dispersed, limiting the three staff members to a small sample size due to time and resources. Additionally, due to the competing demands of research and practice, the research team was not able to prioritize properly conducting inter-rater reliability, which could have served to help further validate the assessment tool. While this was the case, the team did have two to three interviewers for each tool separately conducting the assessment at once so they could be compared to ensure better reliability.

Importantly, El Salvadorian government-employed child care supervisors have felt that the WCI-QCUALS assessments have provided them with information about ways in which childcare centers can improve that each seem in line with the emotional and physical development of children, and which are feasible for improvement. With the ECERS-R, some recommendations that come from the tool to improve the quality of care are not financially feasible and some do not intuitively seem necessary for childhood development needs, such as having temperature controls or child-size toilets. In contrast, identifying the need for more toys that differ in texture, size, and color and can be locally made is feasible, measurable and improves child development. To this end, the government of El Salvador has continued to use the QCUALS until today, 2024.

While this study is limited in the aforementioned ways, data suggest that an instrument developed within and for a low-resource setting is a feasible tool that could be used alone or in combination with others to assess the quality of childcare centers in low-resource settings. This study was meant to generate hypotheses, acknowledging that further assessment tool validation, refining, and testing are needed. With these caveats, the results imply that the WCI-QCUALS, developed in a lower-resource setting to assess daycare centers and residential childcare centers, provided greater variation in assessment ratings than the tool developed for high-resource settings, and in ways that can be associated with meaningful child development outcomes. The two assessment tools have significant overlap in terms of the characteristics they aim to measure and each tool also assesses unique information that the other does not attempt to measure. Raters anecdotally consistently reported that the tool developed for high-resource settings was more “clinical” and rote, whereas the WCI-QCUALS was particularly effective at allowing raters to capture nuances such as the intentionality of caregivers. The high-resource setting assessment tool has a greater emphasis on aspects of childcare centers that require resources more readily available in high-resource settings. Therefore, a setting lacking these resources may be an indication of other intangible aspects of the setting. The WCI-QCUALS instead places a greater focus on the quality of specific caregiver- and staff- interactions and interactions among children. This is a strength of this assessment tool that could lead to more accurate overall assessments of strengths and weaknesses and could point out areas of weakness that are more amenable to change, even in the absence of an influx of new material resources. Therefore, the WCI-QCUALS would be more useful for assisting childcare centers to improve their quality of caregiving.

Another major strength of the WCI-QCUALS is that it integrates multiple data sources systematically to assess indicators of quality: interviews with staff and management within settings, direct observations, and reviews of institution records. In some cases, these data are integrated to gather basic descriptive information (e.g., demographics, basic setting information); in many cases, though, these data are used to gather more in-depth information that guides ratings on multi-faceted indicators (e.g., attachment to caregivers). This strategy allows for a comprehensive approach to assessment and triangulation of data sources in a way that leads to the generation of numerical scores for quantitative analysis. In addition to its purpose as an assessment, the tool also includes the option of providing quality improvement recommendations for childcare settings based on the scores. In this way, the tool generates data and provides direct feedback that can be integrated into efforts to improve quality. Whether or not feedback is used depends on the intent of the WCI-QCUALS user. For instance, in a randomized trial of the intervention strategy, it may not be advisable to use them given concerns about their influence over the results if the administration of the tool is not an explicit part of the intervention.

As the Sustainable Development Goals have requested governments look beyond the survival of children to age five and look toward promoting children thriving in early childhood and beyond, there is growing interest in ways to better measure care for children, including daycare, residential, and school settings (25). In those regards, this study has promising results. When it comes to assessment tools used in lower-resource contexts, too often are the tools designed in and for high-resource settings, without adequately incorporating the local knowledge and cultural context of a given community. Further, these tools are often proprietary and require ongoing payment for use, making them less accessible for lower-resource contexts. Therefore, it is of great interest to contribute to the global conversation on how to contribute to optimal child development within limited resource settings. Empowering governments and childcare providers to have access to non-proprietary tools that accurately and consistently assess the quality of childcare may be beneficial to this end. Such tools can provide meaningful data and appropriate and actionable feedback on aspects of care that can be used to help guide governments and childcare providers toward the improvement of child outcomes. This may also help drive policy decisions related to the most vulnerable populations of children that are informed by evidence. Researchers and childcare providers should continue to develop assessment tools that (1) start with the local resource realities of a given community and context, (2) are appropriately culturally contextualized and seek the collaborative input and centering of local childcare stakeholders, and (3) apply the best knowledge and evidence-informed practices from both the local knowledge context and elsewhere on how to achieve positive outcomes in child development.

## Data Availability

The raw data supporting the conclusions of this article will be made available by the authors, without undue reservation.
